# Building in vitro tools for livestock genomics: chromosomal variation within the PK15 cell line

**DOI:** 10.1186/s12864-023-09931-z

**Published:** 2024-01-11

**Authors:** M. Johnsson, J. M. Hickey, M. K. Jungnickel

**Affiliations:** 1 Department of Animal Breeding and Genetics, Swedish University of Agricultural Sciences, Box 7023, 750 07 Uppsala, Sweden; 2 The Roslin Institute and Royal (Dick) School of Veterinary Studies, The University of Edinburgh, Midlothian, Scotland, EH25 9RG UK

**Keywords:** Pig, Cell lines, PK15, Aneuploidy

## Abstract

**Background:**

Cultured porcine cell lines are powerful tools for functional genomics and in vitro phenotypic testing of candidate causal variants. However, to be utilised for genomic or variant interrogation assays, the genome sequence and structure of cultured cell lines must be realised. In this work, we called variants and used read coverage in combination with within-sample allele frequency to detect potential aneuploidy in two immortalised porcine kidney epithelial (PK15) cell lines and in a pig embryonic fibroblast line.

**Results:**

We compared two PK15 cultured cells samples: a new American Type Culture Collection (ATCC) sample and one that has been utilised and passaged within the laboratory for an extended period (> 10 years). Read coverage and within-sample allele frequencies showed that several chromosomes are fully or partially aneuploid in both PK15 lines, including potential trisomy of chromosome 4 and tetrasomy of chromosome 17. The older PK15 line showed evidence of additional structural variation and potentially clonal variation. By comparison, the pig embryonic fibroblast line was free from the gross aneuploidies seen in the PK15s.

**Conclusions:**

Our results show that the PK15 cell lines examined have aneuploidies and complex structural variants in their genomes. We propose that screening for aneuploidy should be considered for cell lines, and discuss implications for livestock genomics.

**Supplementary Information:**

The online version contains supplementary material available at 10.1186/s12864-023-09931-z.

## Background

A central goal in livestock genetics is the identification of variants and genes responsible for economically important phenotypes/complex traits. Genetic mapping and functional genomic information can be combined with phenotypic data to help identify and prioritise potential causal variants. Cell lines play a critical role in the selection of biologically relevant molecular phenotypic assays. Large-scale epigenomic projects like ENCODE rely heavily on cell lines as models of different cell types, and functional work on causative genes in livestock make use of the relatively few livestock cell lines that exist (e.g. [1]). Two types of cell lines are used for genotype–phenotype studies: immortalised cell lines and Induced Pluripotent Stem Cell (IPSCs) lines. Immortalised cells have been manipulated to proliferate indefinitely and can therefore be cultured without senescing for prolonged periods in vitro (reviewed: [2]). IPSCs, derived from skin or blood cell progenitors, have the capacity to maintain an undifferentiated state indefinitely and be reprogrammed into most cell types in the body [3].

Most CRISPR screens are conducted in vitro. This approach requires an exquisitely precise and detailed knowledge of the genome sequence of target cell lines. It is well established that such sequence details are essential prerequisites to design guide RNAs and direct Cas9 function in CRISPR manipulation and perturbation assays. Less well known is the contribution of genome structure and cell line aneuploidy to CRISPR-genome interrogation. Aneuploidy, in which cells have an abnormal number of chromosomes, has been shown to have a devastating impact on cell phenotype and gene expression [4]. Because of the potential consequences of aneuploidy, widespread aneuploidy of classical cell lines, and the potential for variable karyotype between isolates, we need to characterise the genome of livestock cell lines to enable their use for in vitro variant screening.

For this project, we used short-read sequencing to characterise the genome of PK15, a classic pig cell line established in 1955/1956 from the kidney of an adult Hampshire pig [5, 6], as well as that of a recently isolated pig embryonic fibroblast line for comparison. While this analysis will not resolve the karyotype and structure of the cell line genome, it will detect regions affected by gross abnormalities. We used read coverage in combination with within-sample allele frequency to detect potential aneuploidy in two samples of the PK15 cell line as well as the fibroblast, and re-analysed RNA sequencing data from another two PK15 samples for aneuploidy.

## Results

### Variants detected

We detected a total of 9.0 million single nucleotide variants and 2.8 million insertions/deletions in the PK15 genome, most of which are consistent between the two samples. Table 1 shows crosstabulation of called genotypes between the two sequenced PK15 samples. In most of the variants, the genotype call was consistent between the two samples, with 4.5 million SNPs and 1.0 million indels called as heterozygous and 3.6 million SNPs and 1.1 million indels called as homozygous alternate (that is, homozygous different from the reference genome). In the fibroblast sample, we detected 4.2 million heterozygous SNPs and 1.0 million heterozygous indels, as well as 4.2 million homozygous alternate SNPs and 1.3 million heterozygous alternate indels.

**Table 1:** Crosstable of genotype calls from the two sequenced PK15 samples (the university lab sample in rows and the ATCC sample in columns) for biallelic SNPs and insertions/deletions

*SNPs*				
	PK15 ATCC genotype			
PK15 U.Lab genotype	missing	REF/REF	REF/ALT	ALT/ALT
missing			21,086	39,888
REF/REF			227,593	16,616
REF/ALT	3,128	304,405	4,554,040	216,423
ALT/ALT	10,482	7,046	62,113	3,565,539
*Insertions/deletions*				
	PK15 ATCC genotype			
PK15 U.Lab genotype	missing	REF/REF	REF/ALT	ALT/ALT
missing			35,363	63,242
REF/REF			117,506	36,548
REF/ALT	7,475	59,102	1,036,901	81,673
ALT/ALT	7,810	4,867	44,000	1,143,438

### Depth of read coverage

**Fig. 1:**
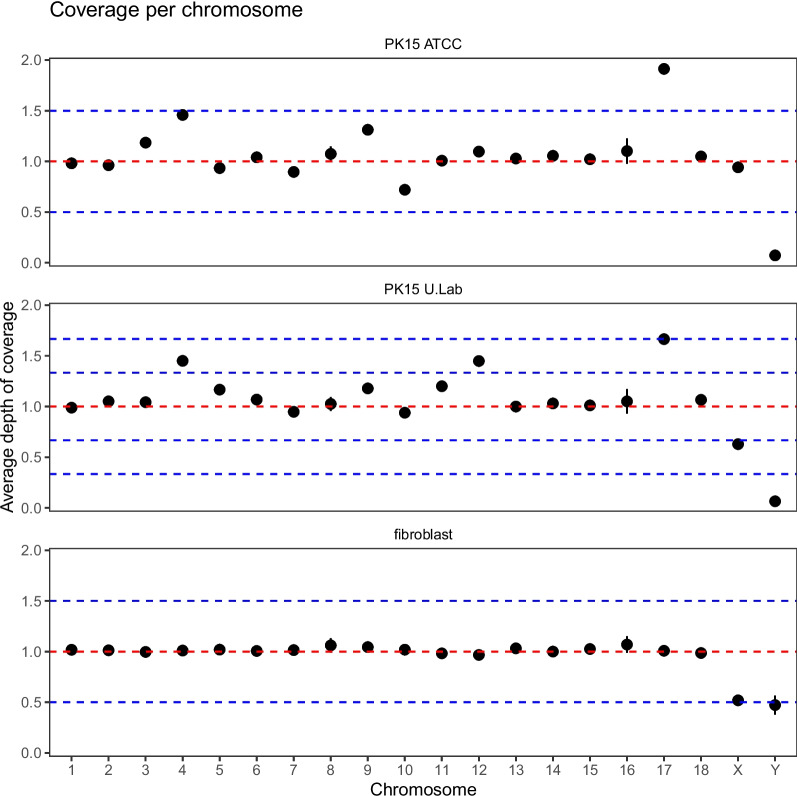
Average depth of coverage on chromosomes 1–18, X and Y in the PK15 and fibroblast genomes. The error bars are plus/minus two times the standard error of the mean. The depth of coverage was standardized by dividing by the median coverage of chromosomes assumed to have the most common copy number (see Methods). The horizontal lines show coverage levels expected for copy numbers from monosomic to trisomic for PK15 ATCC and the fibroblast sample, and coverage levels expected for copy numbers from monosomic to pentasomic for the PK15 U.Lab sample

Differences in the depth of read coverage between chromosomes suggested that several chromosomes are aneuploid in the sequenced PK15 samples. Figure 1 shows the average depth of coverage on each chromosome in the two sequenced PK15 samples and the fibroblast sample. Both the PK15 ATCC and the PK15 university lab sample showed elevated read count in particular on chromosomes 4 and 17 as well as chromosome 9 in the ATCC sample and chromosome 12 in the university lab sample. There was also lower read counts on chromosome 10 in the ATCC sample and in the university lab sample on chromosome X. Both of the PK15 samples had very low coverage on chromosome Y, suggesting that the donor pig was female. There was no evidence of gross aneuploidy in the fibroblast sample, but it showed lower depth of coverage on X and Y, consistent with expectations for a male donor.

**Fig. 2:**
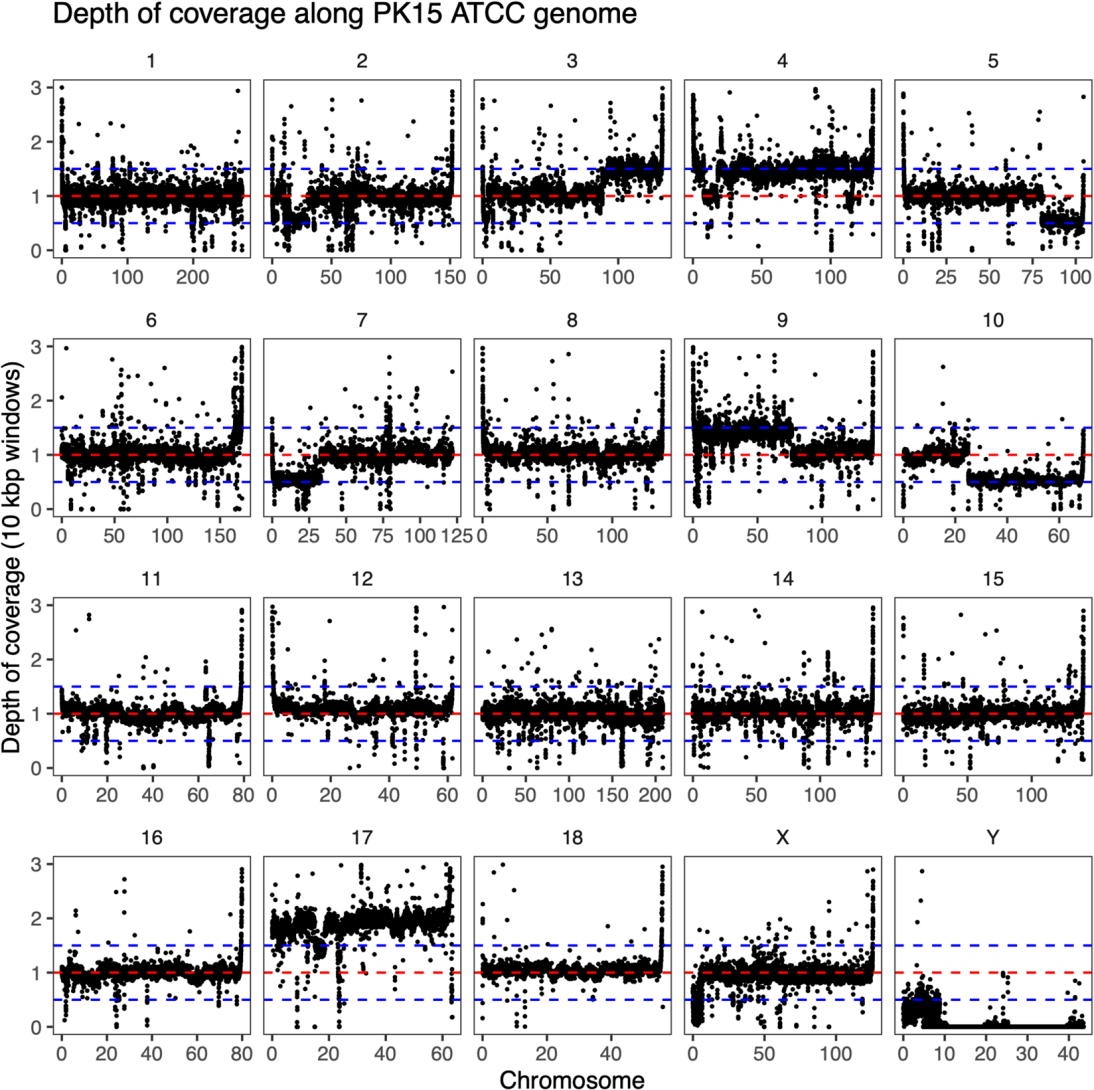
Standardized read coverage in 10 kbp windows along the genome of the PK15 ATCC sample. The depth of coverage was standardized by dividing by the median coverage of chromosomes 1, 2, 3, 6, 13, 14, 15 and 16. The horizontal lines show coverage levels expected for copy numbers from monosomic to trisomic

**Fig. 3:**
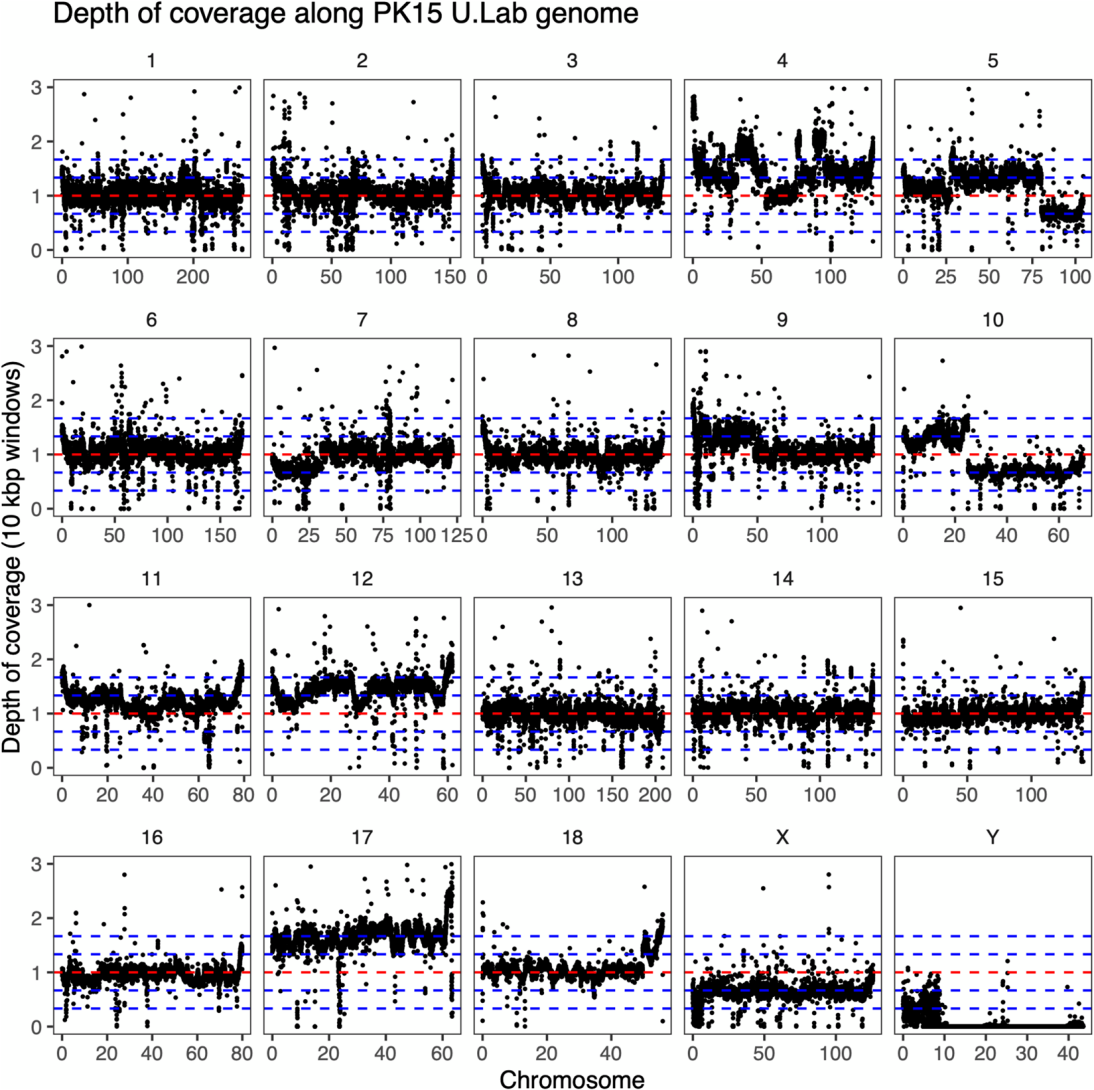
Standardized read coverage in 10 kbp windows along the genome of the PK15 U.Lab sample. The depth of coverage was standardized by dividing by the median coverage of chromosomes 1, 2, 3, 6, 13, 14, 15 and 16. The horizontal lines show coverage levels expected for copy numbers from monosomic to pentasomic

**Fig. 4:**
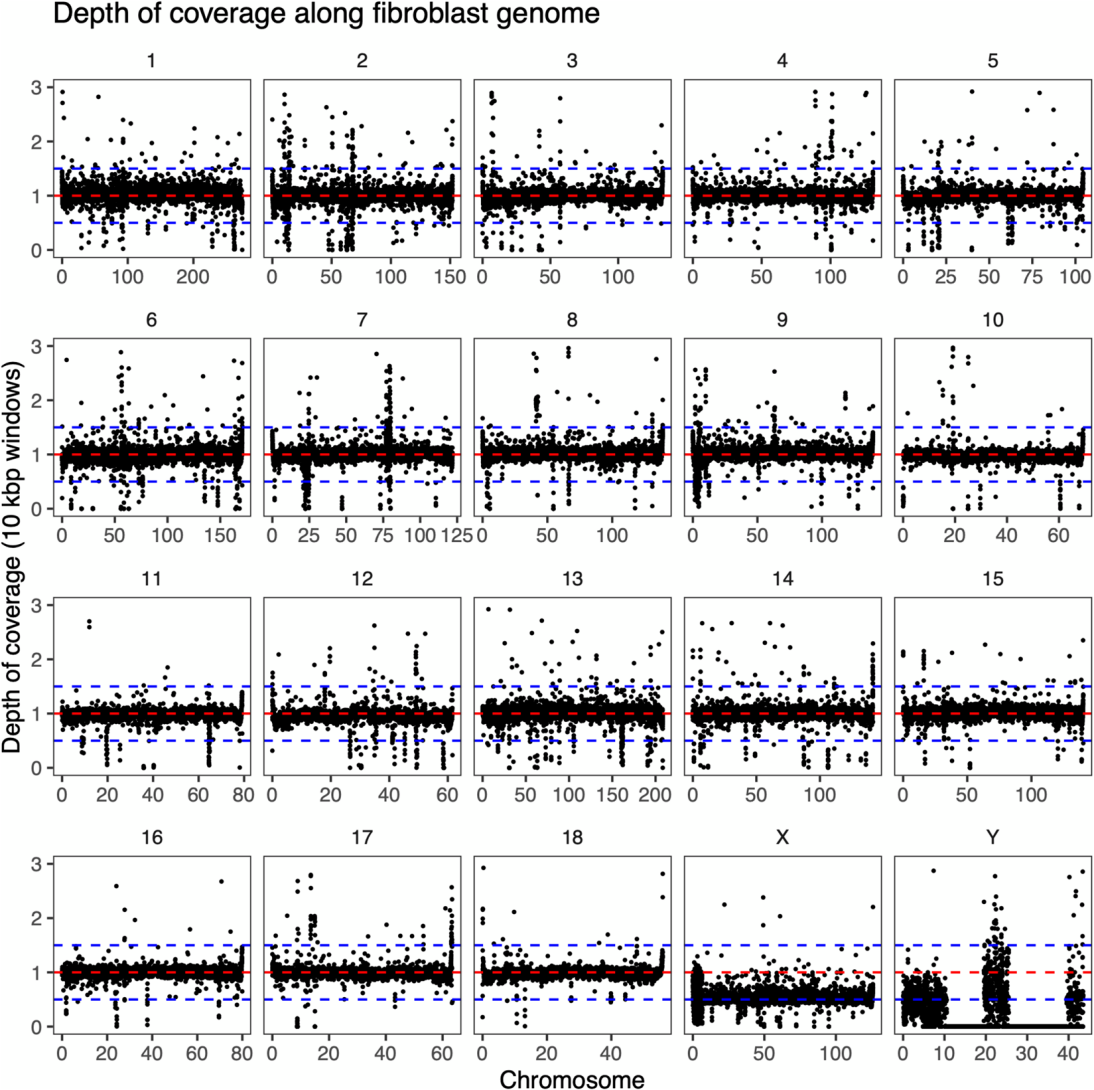
Standardized read coverage in 10 kbp windows along the genome of the fibroblast sample. The depth of coverage was standardized by dividing by the median coverage of chromosomes 1 to 18. The horizontal lines show coverage levels expected for copy numbers from monosmoic to trisomic

Furthermore, variation in the depth of read coverage within chromosomes suggested that there were large-scale structural variants on several chromosomes in the PK15 samples but not the fibroblast sample. Figures 2, 3 and 4 show the average depth of read coverage in 10 kbp windows on each chromosome of the three samples. For example, the two PK15 samples appeared to have large-scale variation in copy numbers on chromosomes 5, 7, 9 and 10 that occurred in broadly the same region. There were also regions with evidence of structural variation in only one sample: chromosome 2 in the ATCC sample, an additional variant on chromosome 5 in the university lab sample, and evidence of complex rearrangements on chromosome 4. There was little evidence of large-scale structural variants in the fibroblast sample; however, the depth of coverage was noisy and smaller-scale structural variation would not be detected in this analysis.

### Whitin-sample allele frequencies

**Fig. 5:**
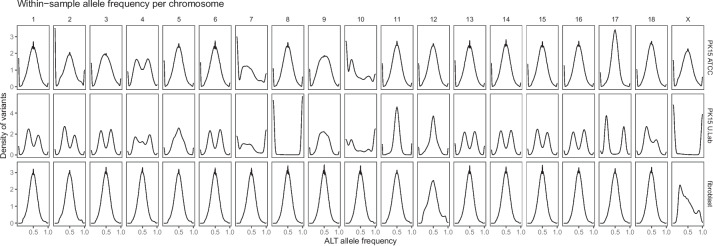
Within-sample allele frequencies at heterozygous sites in genome sequenced cell lines, broken down by chromosome. The density plots show the within-sample allele frequency for the alternative allele, calculated from allelic read counts, for single nucleotide variants that are called heterozygous. In the PK15 samples, sites are included that are heterozygous in at least one of the two samples of the cell line

Within-sample allele frequencies at heterozygous sites confirmed aneuploidy of several chromosomes in the PK15 ATCC sample, and suggested that the PK15 university lab sample was aneuploid for most chromosomes. Figure 5 shows density plots of within-sample allele frequency at sites called as heterozygous in genome sequencing. Most chromosome in the PK15 ATCC sample showed a unimodal distribution with the mode at 0.5, which is what one would expect from a diploid chromosome or a tetrasomic chromosome with a 1:1 allelic relationship. Also consistent with this 1:1 ratio, the allele frequency distribution on putatively tetrasomic chromosome 17 in the ATCC sample was unimodal. In the PK15 university lab sample, most chromosomes showed a multimodal allele frequency distribution, consistent with higher ploidy level with an allelic relationship different from 1:1. Furthermore, chromosomes 8 and X showed an allele frequency distribution where only one allele was present at most sites. In contrast, the fibroblast sample again showed no evidence of aneuploidy.

More specifically, the PK15 university lab sample appeared to be largely trisomic, with allele frequency modes close to 1/3 and 2/3 on chromosomes 1, 2, 3, 6, 13, 14, 15, and 16. Additional figure 1 shows the within-allele frequency densities superimposed on each other, and in relation to the allele frequency modes expected from a diploid and trisomic chromosome. Chromosome 17 in the PK15 university lab sample stood out with allele frequency modes close to 1/5 and 4/5, suggesting a 1:4 allelic relationship. Additional figure 2 shows a comparison with expected allele frequency modes and simulated allele frequency densities from several higher ploidies. Additional text 1 describes the calculations of expected allele frequency modes and simulation of read counts. The observed allele frequency modes could best be explained by a pentasomic chromosome.

### Transcriptome analysis

**Fig. 6:**
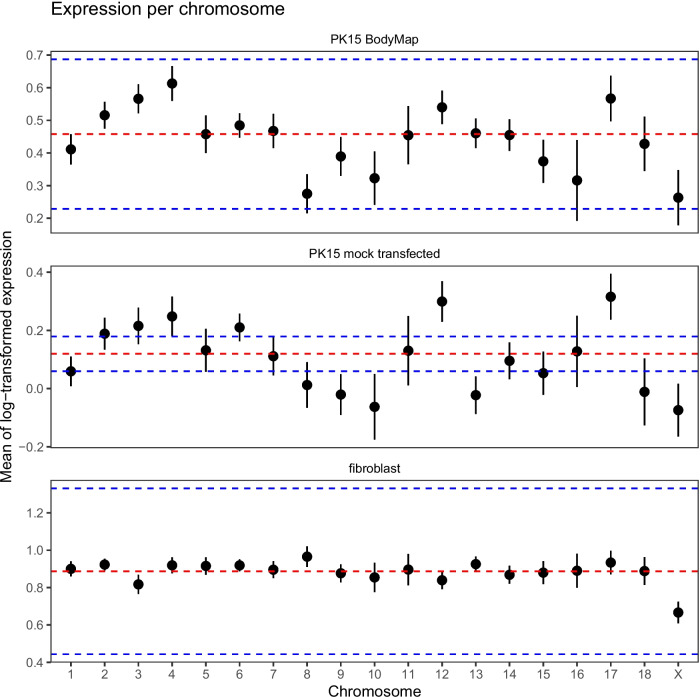
Average expression level in RNA-seq data broken down by chromosome. The red line shows the average of the base-ten logarithm of expression, and the blue lines show 0.5 times and 1.5 times the average. The Y chromosome has been excluded because its average expression is very low in the PK15 samples

Re-analysis of two publicly available PK15 datasets also supported widespread aneuploidy and karyotype variability between isolates of the cell line. Figure 6 shows average expression levels broken down by chromosome in RNA-seq data from two PK15 samples compared to the fibroblast sample. While the fibroblast sample showed relatively uniform expression between the autosomes and reduced expression from the X chromosome, the PK15 samples showed marked chromosome-to-chromosome variation. Both PK15 samples showed relatively higher expression from chromosomes 4, 12, and 17, for example, and relatively low expression from chromosomes 8, 9, and 10.

**Fig. 7:**
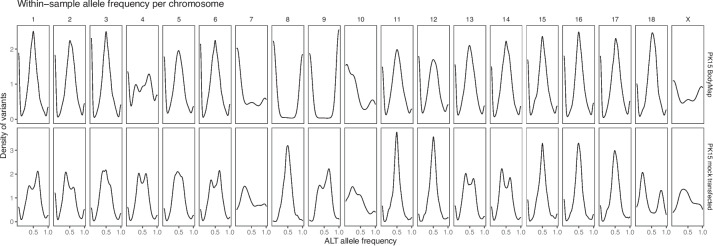
Within-sample allele frequencies at heterozygous sites in RNA-seq data broken down by chromosome. The density plots show the within-sample allele frequency for the alternative allele, calculated from allelic read counts, for single nucleotide variants where both alleles are observed with at least five reads

Within-sample allele frequencies from the PK15 RNA sequencing supported aneuploidy of several chromosomes. Figure 7 shows density plots of within-sample allele frequency at sites called as heterozygous in RNA sequencing. In the PK15 BodyMap sample, most chromosomes had a unimodal distribution with a mode around 0.5, but chromosomes 4, 7, 8, 9, 10 and X deviate. In particular, chromosomes 8 and showed an allele frequency distribution where only one allele was present at most sites, consistent with retention of only one chromosome copy. In the PK15 mock transfected sample, many chromosomes had a bimodal distribution with modes near 1/3 and 2/3, but with variability between chromosomes. Additional figure 3 shows superimposed allele frequency densities from bimodal chromosomes in comparison to the expected allele frequency modes from mixtures of diploid and trisomic cells. These allele frequency modes could be explained by clonal heterogeneity. Furthermore, chromosome 17 showed allele frequency modes close to 1/5 and 4/5, consistent with higher copy number.

## Discussion

Our results show that the PK15 cell lines examined have aneuploidies and complex structural variants in their genomes. Different lineages of the same founder PK15 cell line also showed differences in read coverage and within-sample allele frequencies consistent with structural differences between the genomes of the isolates. By contrast, the newly-derived pig fibroblast cell genome appeared free of such gross abnormalities. These data are consistent with increased instability of cell line karyotype over time [7], as well as observations of extensive aneuploidy in classical cell lines [8–10], including pig kidney cell lines [6, 11]. Our results also similar to the recent results of de Vos et al. [12], who detected aneuploidy in isolates of a pig and a chicken cell line. Their porcine cell line, IPECJ2, showed aneuploidy of several chromosomes, but appears not to be as dramatically disrupted as PK15. Derived in 1989 [13], IPECJ2 is not as old as PK15, and de Vos et al. used an isolate directly from a cell repository.

There is substantial uncertainty about the karyotype of the PK15 samples that cannot be resolved with the available data. In particular, the PK15 university lab sample shows evidence of high copy number of most chromosomes, including ones that have close to the median depth of coverage. This leads us to hypothesise that this sample has gone through a genome-wide triploidisation event, and might thus be polyploid in addition to a number of aneuploidies. However, this hypothesis implies some puzzling consequences: both chromosome 8 and X appear to have only one chromosome copy based on within-sample allele frequencies, but their depths of coverage are higher than expected for monosomic chromosomes. Thus, the same chromosome copy might have been duplicated, with loss of the other chromosome copy, leading to uniparental disomy in the case of X and trisomy in case of chromosome 8. However, alternative explanations are possible, because different mixtures of aneuploid cells can give rise to allele frequency modes and depths of coverage relations that are indistinguishable. For example, a 1:1 mixture of diploid and monosomic cells would give the same patterns as population of fully trisomic cells. Similarly, for the pattern observed on chromosome 17, a 1:1 mixture of diploid and trisomic cells could give the same pattern as a population of fully pentasomic cells. To address these hypotheses, one would need corroborating assays such as ploidy analysis by flow cytometry, karyotyping or fluorescent in situ hybridisation.

Furthermore, while depth of coverage analyses can detect large-scale copy number variation easily, short read sequencing cannot easily answer what type of structural variants cause these deviations. Several chromosomes show evidence of large-scale structural variants, including segmental aneuploidy and complex rearrangements as on chromosome 4 in the PK15 university lab sample. These many be due to segmental duplications and deletions, but may involve translocations to other chromosomes, inversions, or combinations thereof. Resolving the ploidy and structural variation landscape of the PK15 cell line is beyond the scope of this work, and would in any case have to be done on a sample-by-sample basis, given the variation between isolates.

Genome anomalies, such as cell line aneuploidy, have likely only minimally impacted the outcome of over-expression studies frequently used within the molecular genetics field over the last 10–15 years [14]. Overexpression of an individual gene in cell lines may lead to an excess production of the target protein, beyond that of the native protein in wild-type systems. These increased protein levels from gene overexpression are disproportional to allele copy numbers in the genome of the cell line. We believe that this is not a great threat to the validity of studies that have introduced overexpression constructs in PK15, but it may be of greater importance when studying the function of genes and other sequence elements in regions affected by aneuploidy and structural variation within the PK15 genome. With the introduction of CRISPR-based technologies, specifically those that target transcription and gene regulatory function including enhancers, promoters, silencers, (reviewed by [15]), allele-specific gene expression can be precisely targeted and exquisitely modulated within native context. Cell line aneuploidy and chromosomal variation may have a direct impact upon both the efficiency and phenotypic consequence of such assays. As an example of the potential influence of genome aneuploidy on editing efficiency, a genome editing assay with 20% homology-directed repair efficiency will generate repair rates of 4% in diploid and 0.8% in trisomic genome sites respectively.

In addition to impacts on editing efficiency, cell line aneuploidy and clonal variation in copy number will also directly affect the phenotypic outcome of CRISPR gene-editing. Large-scale CRISPR-editing screens in human cells have been performed with haploid cells [16, 17]. As these cells contain only a single copy of the genome, once a mutation is introduced, the cells will display the corresponding phenotype, in these cases often impaired cell growth. In diploid gene regions, CRISPR editing is complicated by the possible outcome of either heterozygous or homozygous edited cells — a heterozygous genotype can mask the effect of any mutations and fail to show a phenotype (reviewed by [18]). In trisomic or tetrasomic genome regions, successful genome editing necessitates concurrent editing of all alleles/copies of a gene in order for a phenotype to be interpretable.

## Conclusions

As we have reported in the current manuscript, a cell line genome can exhibit regions of variable aneuploidy and clonal variation. Given the potential impacts on both editing efficacy and potential phenotype, we suggest that genome ploidy of cell lines be investigated before initiating any type of targeted genome editing interrogation assay. For cell lines that will see extensive use, long-read sequencing or even genome assembly would give the most complete picture of the genome. When that is too expensive, we propose at least performing short read sequencing followed by inspection of depth of coverage and within-sample allele frequency plots.

## Methods

### Cell lines and culture

We sequenced DNA from two Porcine Kidney Epithelial cell (PK15) cultures. One was ordered from ATCC (product name: PK(15) CCL­33), and the other had been in use within the university laboratory for more than 10 years and had undergone an undefined number of passages (which we will refer to as the “university lab” sample). PK15 cell cultures were cultured at 37°C in 5% CO2 in Dulbecco's modified Eagle medium (DMEM; Gibco) supplemented with 10% fetal bovine serum (FBS; Gibco), 1 × non-essential amino acids (NEAA; Gibco) and 1% penicillin–streptomycin (Gibco).

We also sequenced DNA from one culture of Porcine Embryonic Fibroblast (PEFs) cells.

PEFs were isolated from E40 fetuses arising from a mating between a Large White boar carrying a CMV-GFP transgene and a wild-type gilt as described in [19]. The source fetus for the cell line used in this study was phenotypically male. PEFs were maintained in DMEM culture medium supplemented with 10% FBS, 1 mM Sodium Pyruvate (Gibco), 2 mM L-Glutamine (Gibco), 1 × NEAA, 0.1 nm β-mercaptoethanol (Gibco). Cells were plated at a density of 1 × 10^4^/cm^2^, fed every second day and passaged every 3–4 days.

### Genome sequencing

Genomic DNA was prepared from cell lines with the DNAeasy Blood & Tissue kit (Qiagen). We prepared one library for each cell line. The ATCC PK15 sample and the fibroblast sample were sequenced by GeneWiz (Essex, UK) on the Illumina NovaSeq platform. The university lab PK15 sample was sequenced by Edinburgh Genomics (University of Edinburgh, UK) on the Illumina HiSeq X platform. Both used Illumina TruSeq libraries with 2 × 150 bp paired-end reads. The total number of mapped reads are shown in Additional Table 1. They add up to approximately 56X coverage of the pig genome for the PK15 university laboratory sample, 29X coverage for the PK15 ATCC sample, and 34X coverage for the fibroblast sample.

Reads were aligned to the Sscrofa11.1 reference genome [20] using BWA MEM [21] after trimming with Trimmomatic version 0.36 [22]. Alignment was followed by duplicate removal with Picard version 2.9.0 (http://broadinstitute.github.io/picard), and base quality score recalibration using GATK 3.5 [23]. For the PK15 samples, we performed base quality score recalibration by bootstrapping. That is, by first calling a preliminary set of variants, and using it as input to the GATK base quality score recalibration tool, and then calling a final set of variants using the recalibrated alignments. For the fibroblast sample, we used variant positions from the Ensembl Variant database for base quality score recalibration.

We called variants using GATK’s gVCF workflow, using the HaplotypeCaller followed by joint genotyping with GenotypeGVCFs [24]. The two PK15 samples were called jointly, whereas the fibroblast sample was called separately. We then performed hard filtering using thresholds of quality by depth QD < 2.0, Fisher strand score FS > 60.0, root mean square mapping quality MQ < 40.0, mapping quality rank sum tests core MQRankSum < -12.5 and read position rank sum score ReadPosRankSum < -8.0 for single nucleotide variants and QD < 2.0, FS > 200.0 and ReadPosRankSum < -20.0 for insertion/deletions.

### RNA sequencing

Standard RNA-Seq was carried out for the porcine fibroblast cell line. Total RNA was extracted from fibroblast cells using Qiagen Maxi RNeasy (Qiagen, 75162) following the manufacturer’s protocol including the on-column DNase digestion. The RNA sample was quantified with the Qubit dsRNA broad range kit (Thermofisher) and was quality checked with the Agilent bioanalyzer 2100 (RNA integrity number of 8.9). Libraries were then prepared and sequencing using the Illumina NovoSeq platforms, 2 × 150 bp configuration and ~ 20 M paired-end reads per sample (Genewiz-Azenta; Essex, UK).

For PK15, we downloaded two publicly available RNA-seq datasets (sample accession SAMN15150297 from [25] and a mock-transfected PK15 sample from project accession PRJNA436951). We regarded the three replicate runs of the mock-transcripted PK15 sample as technical replicates of the same sample.

We quantified gene expression with Kallisto version 0.44.0 [26] against the pig transcriptome (Ensembl Gene database version 107). We calculated gene expression averages per chromosome as the average of the base-ten logarithm of the TPM expression values from Kallisto, including transcripts with greater than zero reads observed. The total number of pseudoaligned reads are shown in Additional Table 1.

We also called variants from PK15 RNA-seq reads using STAR version 2.7.8a [27]. We marked duplicate reads with Picard version 2.9.0 and performed base quality score recalibration with GATK version 4.3.0.0, with the variant positions detected from PK15 genome sequencing. We applied the GATK SplitNCigarReads tool to split alignment of reads with long gaps, and then called variants using GATK’s gVCF workflow. As recommended by the GATK workflows for variant calling from RNA-seq, we used a minimum quality threshold for variant calling of 20, and filtered the resulting variants using the thresholds FS > 30.0 || QD < 2.0 and filtering clusters of three or more variants in windows of 35 bp.

### Aneuploidy detection

In order to detect large-scale copy number variation and aneuploidy, we divided the pig genome chromosomes 1–18, X and Y into 10 kbp windows, and counted the number of genome sequencing reads mapping in each window with BEDTools version 2.26.0 [28], after removing reads marked as duplicate by Picard.

In order to estimate the allele frequencies within each sample, we extracted the allelic depth for each biallelic single nucleotide variant with BCFtools version 1.10.2 [29]. From these allelic depths, we calculated the observed frequency of the alternative allele within each library for variants that were called as heterozygote, i.e., show evidence for reads with both alleles. Because the variants in the PK15 samples were called jointly, we included variants that were called heterozygous in either sample. The variants fibroblast sample were called with only that sample, and thus only sites called as heterozygous within that sample were included.

In order to estimate the allele frequencies within the RNA sequence samples, we extracted allelic depths with BCFtools and calculated allelic depths from single nucleotide variants as above. Because the variation in read coverage is much more dramatic in RNA-seq than in genome sequencing, we included only positions where at least five reads were observed from each allele either in the PK15 BodyMap sample or the mock-transfected PK15 sample.

### Supplementary Information

**Additional file 1.** Mapping statistics. Number of reads mapped, or pseudoaligned, for whole-genome and RNA sequencing sampled analysed in this study.

**Additional file 2.** Simulation of expected allele frequencies under aneuploidy.

**Additional file 3.** Superimposed within-sample allele frequencies.

**Additional file 4.** Expected versus observed within-sample allele frequencies PK15 U.Lab chromosome 17.

**Additional file 5:** Expected versus observed within-sample allele frequencies PK15 BodyMap.

## Data Availability

The sequence data generated in this work has been uploaded to the European Nucleotide Archive (https://www.ebi.ac.uk/ena/) with project accession PRJEB60951. This consists of whole-genome resequencing of two PK15 isolates and one porcine embryonic fibroblast line, and RNA sequencing of the porcine embryonic fibroblast line. We also used publicly available RNA-seq data from sample SAMN15150297 and project PRJNA436951. The coverage and allelic depth results have been deposited to Fishare with digital object identifier 10.6084/m9.figshare.24599430.

## References

[1] Walker LR, Engle TB, Vu H, Tosky ER, Nonneman DJ, Smith TP (2018). Synaptogyrin-2 influences replication of Porcine circovirus 2. Plos Genet.

[2] Irfan Maqsood M, Matin MM, Bahrami AR, Ghasroldasht MM (2013). Immortality of cell lines: challenges and advantages of establishment. Cell Biol Int.

[3] Zakrzewski W, Dobrzyński M, Szymonowicz M, Rybak Z (2019). Stem cells: past, present, and future. Stem Cell Res Ther.

[4] Sheltzer JM, Torres EM, Dunham MJ, Amon A (2012). Transcriptional consequences of aneuploidy. Proc Natl Acad Sci.

[5] Harris M (1959). Growth measurements on monolayer cultures with an electronic cell counter. Can Res.

[6] Ruddle FH (1961). Chromosome variation in cell populations derived from pig kidney. Can Res.

[7] Duesberg P, Rausch C, Rasnick D, Hehlmann R (1998). Genetic instability of cancer cells is proportional to their degree of aneuploidy. Proc Natl Acad Sci.

[8] Deaven LL, Petersen DF (1973). The chromosomes of CHO, an aneuploid Chinese hamster cell line: G-band, C-band, and autoradiographic analyses. Chromosoma.

[9] Adey A, Burton JN, Kitzman JO, Hiatt JB, Lewis AP, Martin BK (2013). The haplotype-resolved genome and epigenome of the aneuploid HeLa cancer cell line. Nature.

[10] Macville M, Schröck E, Padilla-Nash H, Keck C, Ghadimi BM, Zimonjic D (1999). Comprehensive and definitive molecular cytogenetic characterization of hela cells by spectral karyotyping. Can Res.

[11] Pirtle EC (1966). Variation in the modal chromosome number of two PK-15 porcine kidney cell lines. Am J Vet Res.

[12] de Vos J, Crooijmans RPMA, Derks MFL, Kloet SL, Dibbits B, Groenen MAM (2023). Detailed molecular and epigenetic characterization of the pig IPECJ2 and chicken SL-29 cell lines. IScience.

[13] Schierack P, Nordhoff M, Pollmann M, Weyrauch KD, Amasheh S, Lodemann U (2006). Characterization of a porcine intestinal epithelial cell line for in vitro studies of microbial pathogenesis in swine. Histochem Cell Biol.

[14] Prelich G (2012). Gene overexpression: uses, mechanisms, and interpretation. Genetics.

[15] Akinci E, Hamilton MC, Khowpinitchai B, Sherwood RI (2021). Using CRISPR to understand and manipulate gene regulation. Development.

[16] Findlay GM, Boyle EA, Hause RJ, Klein JC, Shendure J (2014). Saturation editing of genomic regions by multiplex homology-directed repair. Nature.

[17] Findlay GM, Daza RM, Martin B, Zhang MD, Leith AP, Gasperini M (2018). Accurate classification of BRCA1 variants with saturation genome editing. Nature.

[18] Yin Z, Chen L (2017). Simple meets single: the application of CRISPR/Cas9 in Haploid embryonic stem cells. Stem Cells Int.

[19] Thomson AJ, Pierart H, Meek S, Bogerman A, Sutherland L, Murray H (2012). Reprogramming pig fetal fibroblasts reveals a functional LIF signaling pathway. Cell Reprogr.

[20] Warr A, Affara N, Aken B, Beiki H, Bickhart DM, Billis K, et al. An improved pig reference genome sequence to enable pig genetics and genomics research. GigaScience. 2020;9:giaa051. 10.1093/gigascience/giaa051 PMC7448572 32543654

[21] Li H. Aligning sequence reads, clone sequences and assembly contigs with BWA-MEM. arXiv preprint arXiv:13033997. 2013. 10.48550/arXiv.1303.3997.

[22] Bolger AM, Lohse M, Usadel B (2014). Trimmomatic: a flexible trimmer for Illumina sequence data. Bioinformatics.

[23] McKenna A, Hanna M, Banks E, Sivachenko A, Cibulskis K, Kernytsky A (2010). The genome analysis Toolkit: a MapReduce framework for analyzing next-generation DNA sequencing data. Genome Res.

[24] Poplin R, Ruano-Rubio V, DePristo MA, Fennell TJ, Carneiro MO, Van der Auwera GA, et al. Scaling accurate genetic variant discovery to tens of thousands of samples. BioRxiv. 2018:201178. 10.1101/201178.

[25] Jin L, Tang Q, Hu S, Chen Z, Zhou X, Zeng B (2021). A pig BodyMap transcriptome reveals diverse tissue physiologies and evolutionary dynamics of transcription. Nat Commun.

[26] Bray NL, Pimentel H, Melsted P, Pachter L (2016). Near-optimal probabilistic RNA-seq quantification. Nat Biotechnol.

[27] Dobin A, Davis CA, Schlesinger F, Drenkow J, Zaleski C, Jha S (2013). STAR: ultrafast universal RNA-seq aligner. Bioinformatics.

[28] Quinlan AR (2014). BEDTools: the Swiss-army tool for genome feature analysis. Curr Protoc Bioinformatics.

[29] Danecek P, Bonfield JK, Liddle J, Marshall J, Ohan V, Pollard MO (2021). Twelve years of SAMtools and BCFtools. GigaScience.

