# Supplementary material for: Building in vitro tools for livestock genomics: chromosomal variation within the PK15 cell line
Source: BMC Genomics. 2024 Jan 11;25:49. doi: 10.1186/s12864-023-09931-z (PMC10782621; doi:10.1186/s12864-023-09931-z)
Supplement: Supplementary file 1 — Additional file 1. Mapping statistics. Number of reads mapped, or pseudoaligned, for whole-genome and RNA sequencing sampled analysed in this study. [file 12864_2023_9931_MOESM1_ESM.pdf]

Additional table 1. Mapping statistics. Number of reads mapped, or pseudoaligned, for whole-genome and RNA sequencing sampled analysed in this study.

Sample name	Type	Accession number	Reads mapped (bwa)	Reads mapped (STAR)	Fragments pseudoaligned (Kallisto)
PK15 U.Lab	WGS	ERS14853348	935 565 221		
PK15 ATCC	WGS	ERS14853347	489 397 868		
fibroblast	WGS	ERS14853349	571 630 542		
fibroblast	RNA-seq	ERS14853349			26 646 862
PK15 BodyMap	RNA-seq	PRJNA436951		52 551 038	16 137 376
PK15 mock	RNA-seq	SAMN15150297		216 275 710	99 820 799
