# Supplementary material for: Building in vitro tools for livestock genomics: chromosomal variation within the PK15 cell line
Source: BMC Genomics. 2024 Jan 11;25:49. doi: 10.1186/s12864-023-09931-z (PMC10782621; doi:10.1186/s12864-023-09931-z)
Supplement: Supplementary file 2 — Additional file 2. Simulation of expected allele frequencies under aneuploidy. [file 12864_2023_9931_MOESM2_ESM.pdf]

Simulation of expected allele frequencies under aneuploidy

We simulated within-sample allele frequencies from different copy numbers in order to compare the observed frequencies with expectations. Assuming a given copy number and possible ratios between alternative and reference alleles, we calculated the expected allele frequency modes. For example, a monosomic chromosome allows only 1 or 0 alleles, and thus frequency modes at 0 and 1; a diploid chromosome allows 0, 1 or 2 alleles and modes at 0, 0.5 and 1; and generally, modes $(0, \frac{1}{p}, \frac{2}{p}, \ldots, \frac{p-1}{p}, 1)$ for a chromosome with p copies.

All modes might not be possible. First, sites with within-sample frequencies of 0 and 1 can only be called in PK15 samples if the other sample has both alleles, for example a chromosome that is normally diploid but has lost a copy in one of the samples. Second, because only heterozygous sites are included, the modes at 0 and 1 would be excluded for any chromosome with higher copy number than monosomic. Third, higher copy number chromosomes may exist only in particular ratios depending on what kind of mutation they arose from. For example, a tetrasomic chromosome arising from failure of segregation of diploid chromosomes would be expected to have an allelic ratio of 1:1 and a frequency mode at 0.5.

For mixed cell populations with different copy number, we consider a number of subpopulations with p_i copies of the chromosome each and subpopulation frequency f_i. We weight the number of alleles contributed by each subpopulation by the frequency of the population, generate all possible combinations of alleles, and divide by the average number of chromosomes per cell $\sum_i (p_i f_i)$.

After calculating the allele frequency modes for a particular copy number, we simulated allelic read counts for 10,000 sites. First, we drew the depth of coverage for a simulated site from a Poisson distribution $r_l \sim Po(40)$ with mean 40 representing the average coverage of the PK15 U.Lab sample. We uniformly randomly sampled a frequency mode f out of the possible modes. We then drew alternative allele counts for the site from a Binomial distribution with $r_{ALT} \sim Binom(2, f)$, with two trials representing two alleles and success probability equaling the expected frequency of the mode. We calculated the observed frequency from the simulated read counts and plotted the densities in a similar way as the real observed within-sample frequencies.
