# Supplementary material for: Building in vitro tools for livestock genomics: chromosomal variation within the PK15 cell line
Source: BMC Genomics. 2024 Jan 11;25:49. doi: 10.1186/s12864-023-09931-z (PMC10782621; doi:10.1186/s12864-023-09931-z)
Supplement: Supplementary file 3 — Additional file 3. Superimposed within-sample allele frequencies. [file 12864_2023_9931_MOESM3_ESM.pdf]

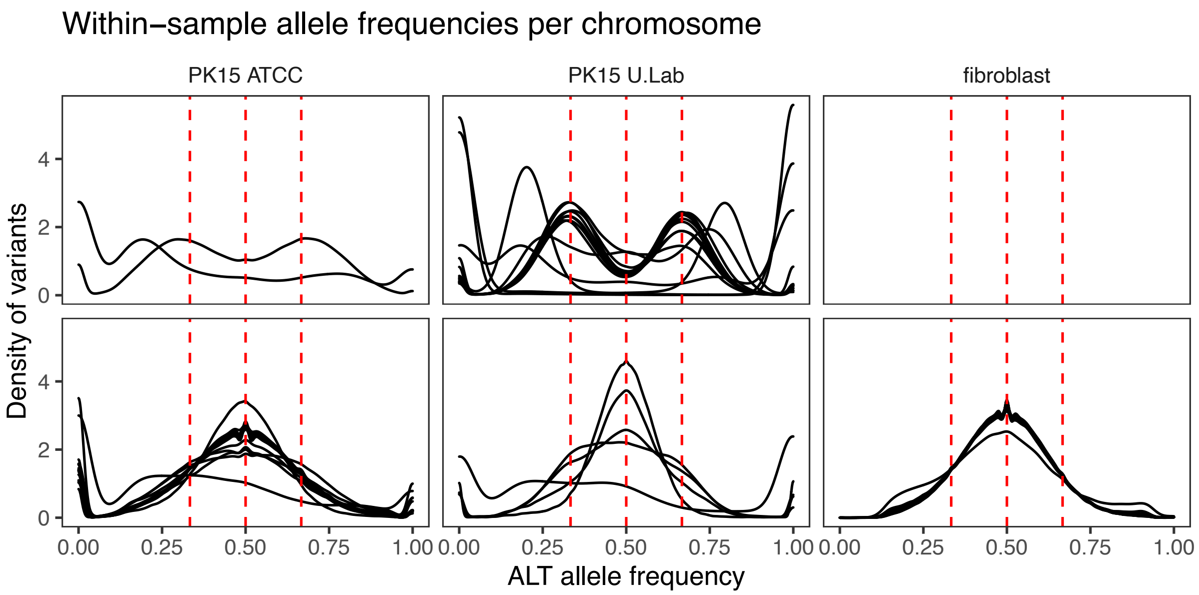

Additional figure 1. Superimposed within-sample allele frequencies. Comparison of within-sample allele frequencies with expected allele frequency modes in a diploid or trisomic sample.
