# Supplementary material for: Building in vitro tools for livestock genomics: chromosomal variation within the PK15 cell line
Source: BMC Genomics. 2024 Jan 11;25:49. doi: 10.1186/s12864-023-09931-z (PMC10782621; doi:10.1186/s12864-023-09931-z)
Supplement: Supplementary file 4 — Additional file 4. Expected versus observed within-sample allele frequencies PK15 U.Lab chromosome 17. [file 12864_2023_9931_MOESM4_ESM.pdf]

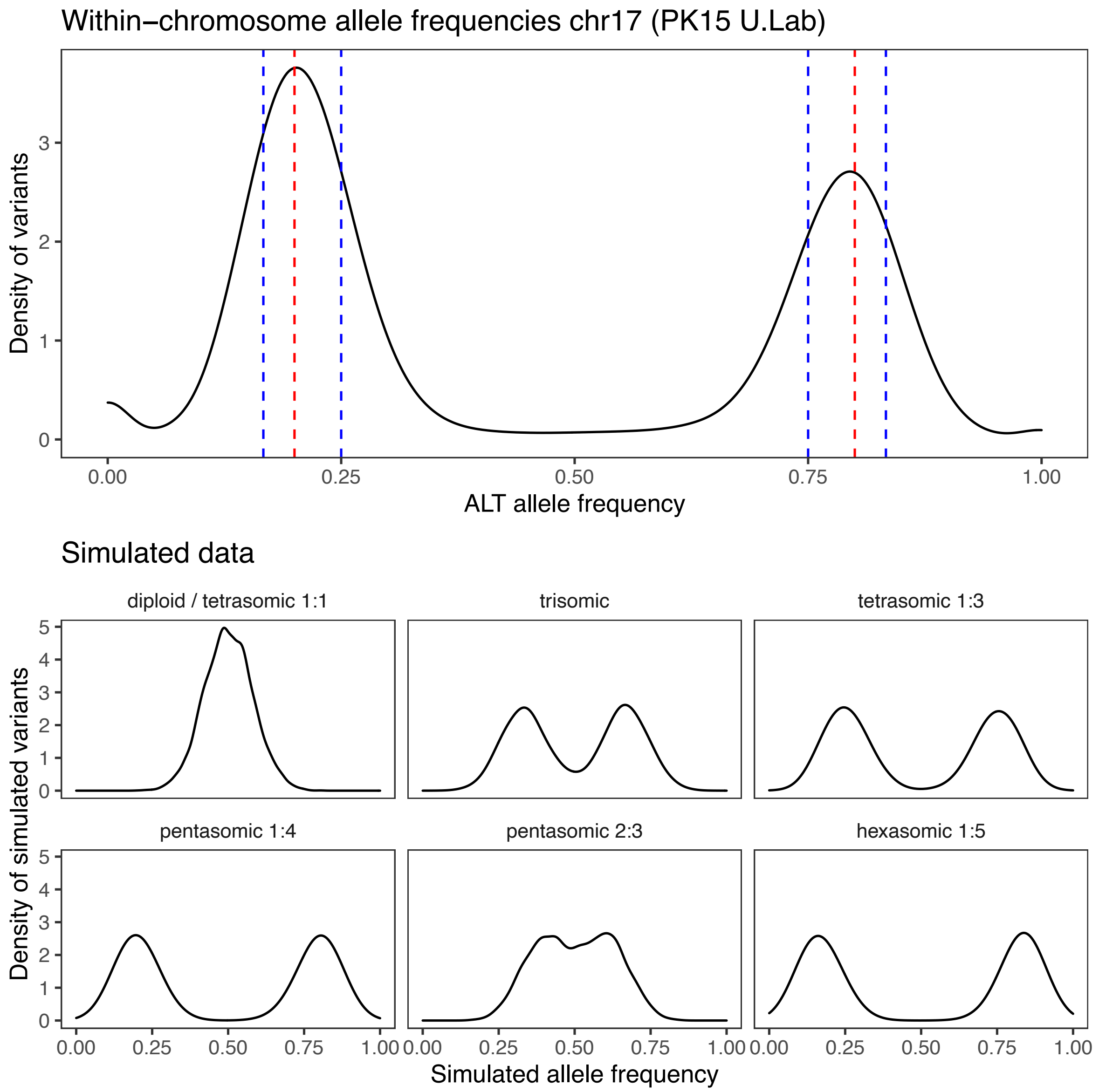

Additional figure 2. Expected versus observed within-sample allele frequencies PK15 U.Lab chromosome 17. Comparison of within-sample allele frequencies on PK15 U.Lab chromosome 17 with simulated allele frequencies. The red lines show the expected allele frequency modes for a pentasomic chromosome with a 1:4 ratio of alleles, whereas the blue lines show expected allele frequency modes for a tetrasomic chromosome (1:3 ratio of alleles) and a hexasomic chromosome (1:5 ratio of alleles).
