# Supplementary material for: Building in vitro tools for livestock genomics: chromosomal variation within the PK15 cell line
Source: BMC Genomics. 2024 Jan 11;25:49. doi: 10.1186/s12864-023-09931-z (PMC10782621; doi:10.1186/s12864-023-09931-z)
Supplement: Supplementary file 5 — Additional file 5: Expected versus observed within-sample allele frequencies PK15 BodyMap. [file 12864_2023_9931_MOESM5_ESM.pdf]

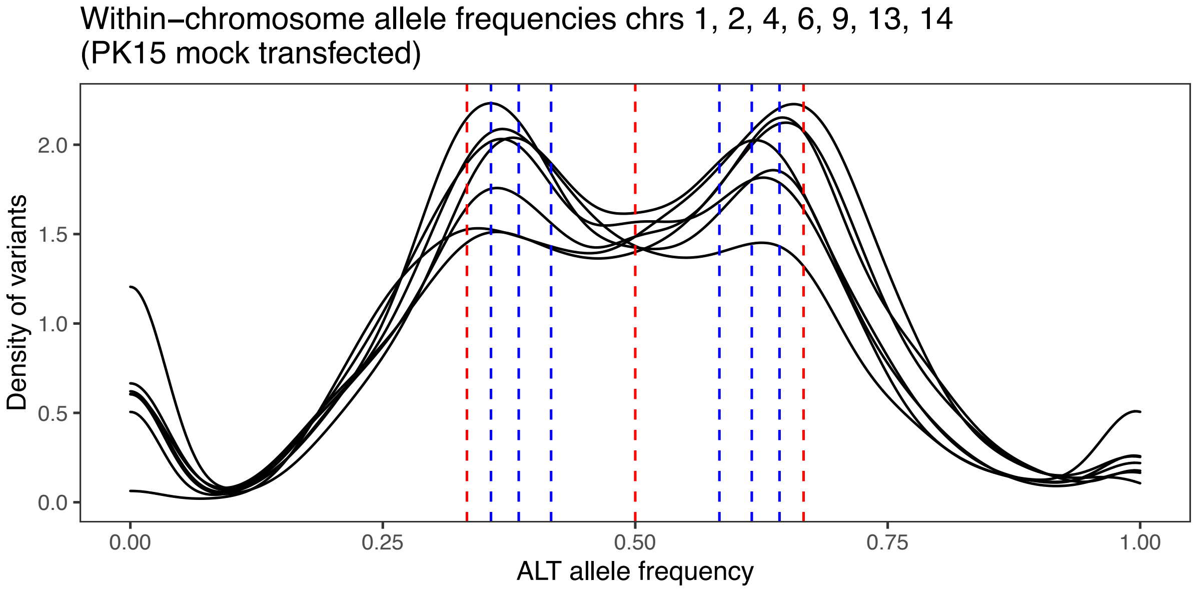

Additional figure 3. Expected versus observed within-sample allele frequencies PK15 BodyMap. Comparison of within-sample allele frequencies on potentially trisomic chromosomes of the PK15 BodyMap sample with expected allele frequency modes. The red lines show the expected allele frequency modes of purely diploid and trisomic chromosomes, and the blue lines show expected modes from mixtures of diploid and trisomic chromosomes with 80%, 60% and 40% trisomic cells.
